# Development and Performance Evaluation of a Temperature- and Salt-Resistant Bio-Based Profile-Control and Oil Displacement System

**DOI:** 10.3390/polym18141768

**Published:** 2026-07-20

**Authors:** Xianglong Yu, Baoshan Guan, Lixin Huang, Yilin Xin, Kaiqi Leng, Jianlong Xiu

**Affiliations:** 1 School of Engineering Science, University of Chinese Academy of Sciences, Beijing 100049, China; yuxianglong23@mails.ucas.ac.cn (X.Y.); gbs7611@163.com (B.G.); huanglixin69@petrochina.com.cn (L.H.); xinyilin24@mails.ucas.ac.cn (Y.X.); lengkaiqi20@mails.ucas.ac.cn (K.L.); 2 Institute of Porous Flow and Fluid Mechanics, Chinese Academy of Sciences, Langfang 065007, China; 3 PetroChina Research Institute of Petroleum Exploration and Development, Beijing 100083, China

**Keywords:** biomass, scleroglucan, green profile control and flooding, temperature and salt resistance, enhanced oil recovery (EOR)

## Abstract

High-temperature and high-salinity reservoirs (typically referring here to temperatures ≥ 100 °C and salinities > 100 g/L) impose stringent requirements on chemical flooding and profile-control agents, particularly in terms of thermal stability, salt tolerance, injectivity, and environmental compatibility. In this study, a bio-based composite mobility-control and oil-displacement system was developed by combining carbonized corn-straw particles with the biopolymer scleroglucan. Corn-straw biomass particles were prepared by pyrolysis at 500 °C followed by ball milling for 2 h. Their particle-size distribution, elemental composition, and suspension stability were characterized, and the rheological behavior, thermal and salt tolerance, long-term aging stability, injectivity, plugging performance, and enhanced-oil-recovery efficiency of the composite system were evaluated systematically. The average particle size decreased from 25.6 μm for mechanically ground straw to 2.8 μm after carbonization and ball milling. The H/C atomic ratio of the carbonized particles was 0.31, indicating enhanced aromatization and structural stability. A scleroglucan concentration of 1000 ppm provided a suspension rate of 97%, balancing suspension stability and chemical dosage. The composite system maintained stable viscosity and viscoelasticity from 30 to 130 °C in deionized water, saturated NaCl solution, and saturated CaCl_2_ solution, with viscosity loss below 10%. After sealed anaerobic aging at 100 °C for 28 days, the viscosity retention remained above 90%. Sand-pack tests showed stable injectivity in media with permeabilities of 1235 and 2064 mD and a plugging efficiency of 95.7% in a 2846 mD model. In oil-displacement experiments, the composite system increased the final recovery factor from 46.6% for scleroglucan flooding alone to 53.3%, corresponding to an additional 6.7 percentage points. These results demonstrate that the carbonized biomass particle-scleroglucan system has promising thermal stability, salt tolerance, plugging capacity, and oil-displacement performance, providing a potential green strategy for mobility control in harsh reservoir environments.

## 1. Introduction

Many mature oilfields have entered the middle-to-late stage of development, in which long-term water flooding has intensified reservoir heterogeneity and promoted the formation of dominant flow channels. These channels accelerate water breakthrough, reduce volumetric sweep efficiency, and leave a substantial fraction of residual oil in poorly swept regions [1,2,3,4]. Profile control and water-shutoff technologies are therefore important approaches for improving sweep efficiency and stabilizing production in high-water-cut reservoirs [5,6]. Although conventional polymer flooding and chemical plugging systems have been widely applied, many synthetic polymers and plugging agents still show insufficient resistance to high temperature and high salinity, limited adaptability to complex pore structures, and relatively high environmental burdens [7]. These limitations have stimulated interest in low-cost, bio-based, and reservoir-adaptive systems for enhanced oil recovery (EOR). Polymer flooding improves oil recovery mainly by increasing the viscosity of the injected aqueous phase, thereby reducing the mobility ratio between the displacing fluid and crude oil and improving volumetric sweep efficiency [8]. Profile-control and water-shutoff treatments are designed to selectively reduce the permeability of dominant flow channels through gel formation, particle retention, mechanical bridging, adsorption, or pore-throat plugging, forcing subsequent injected water to enter previously unswept or poorly swept regions. In particle–polymer composite systems, the polymer phase provides viscosity enhancement and particle-carrying capacity, whereas the dispersed particles contribute to pore-throat bridging, retention, and flow diversion. Therefore, the combined regulation of polymer concentration, particle size, suspension stability, viscoelasticity, and long-term thermal–salinity stability is essential for achieving effective mobility control in heterogeneous reservoirs [9,10,11,12].

Bio-based polymers have attracted increasing attention in profile control and oil-displacement applications because of their renewable origin, biodegradability, and relatively low environmental burden. Commonly investigated bio-based polymers include xanthan gum, guar gum, cellulose derivatives, starch derivatives, alginate, and scleroglucan. Xanthan gum shows good viscosity-building ability and salt tolerance, but its long-term stability may be affected under severe thermal and biological conditions [13]. Guar gum and starch-based polymers are low-cost and readily available, but they may suffer from poor thermal stability and microbial degradation [14]. Cellulose derivatives can provide improved viscosity and environmental compatibility, whereas their solubility and injectivity need to be carefully controlled. Compared with these bio-based polymers, scleroglucan is characterized by a relatively rigid triple-helical structure and weak ionic sensitivity, making it a promising candidate for mobility control in high-temperature and high-salinity reservoirs.

Scleroglucan is an extracellular β-glucan produced by Sclerotium species and is characterized by a relatively rigid triple-helical conformation. Compared with charge-sensitive synthetic polymers, its weakly ionic or nonionic character helps improve its tolerance to saline environments, while its molecular rigidity and hydration capacity contribute to favorable thickening and viscoelastic behavior. Previous studies have shown that biopolymers, including scleroglucan and related polysaccharides, can maintain relatively stable viscosity under harsh reservoir conditions and are therefore promising candidates for mobility control in high-temperature and high-salinity reservoirs [15,16].

Previous studies have demonstrated that scleroglucan solutions can effectively increase water viscosity and maintain relatively stable rheological properties under high-salinity conditions. Because of its rigid triple-helical conformation, scleroglucan is less sensitive to ionic shielding than many charged synthetic polymers. In addition to single-polymer systems, particle–polymer composite systems have also been explored to improve suspension stability, plugging strength, and resistance to shear-induced structural breakdown [17]. However, most reported particle-reinforced systems have used inorganic particles, modified silica, clay minerals, or synthetic polymer particles. The combination of scleroglucan with carbonized biomass particles for simultaneous water thickening, particle suspension, profile control, and oil displacement remains insufficiently studied [18].

Agricultural straw is an abundant lignocellulosic waste resource with advantages of wide availability, low cost, and renewability [19]. After carbonization, grinding, or surface modification, straw-derived particles can act as dispersed plugging or reinforcing materials in oilfield profile-control systems. Earlier studies have prepared profile-control agents using corn straw, rice straw, wheat straw, and related biomass-derived particles, demonstrating their potential to improve plugging capacity and regulate fluid flow in porous media [20,21]. However, the combined use of carbonized straw particles and salt-tolerant biopolymers for high-temperature and high-salinity oil-displacement systems remains insufficiently investigated, especially with respect to suspension stability, rheological response, injectivity, and pore-scale plugging behavior. Biomass-derived particles, including straw powder, cellulose particles, lignin-based particles, and biochar particles, have been investigated as low-cost plugging or reinforcing materials for profile control. These particles can improve flow resistance through mechanical bridging, adsorption, and pore-throat plugging. However, the direct use of carbonized corn-straw particles in combination with scleroglucan for high-temperature and high-salinity profile control and oil displacement has rarely been reported. Therefore, the present study aims to fill this gap by evaluating the particle size, suspension stability, rheological response, injectivity, plugging performance, and oil-displacement efficiency of a carbonized corn-straw particle–scleroglucan composite system [22].

For a particle–polymer profile-control system, several key properties determine its applicability in reservoirs. Particle size controls the matching degree between particles and pore throats, thereby affecting injectivity and deep migration. Suspension stability determines whether particles can remain uniformly dispersed during injection; insufficient suspension stability may cause sedimentation, near-wellbore plugging, and unstable profile-control performance [23]. Apparent viscosity and viscoelastic parameters, including storage modulus *G*′ and loss modulus *G*″, are directly related to mobility control, particle carrying capacity, and resistance to structural disruption under shear. In addition, long-term viscosity retention under high-temperature and high-salinity conditions is necessary for maintaining effective flow control in harsh reservoirs. Finally, injectivity, plugging efficiency, and oil-displacement efficiency are essential application-oriented indicators for evaluating whether the system can enter porous media, selectively block high-permeability channels, and improve oil recovery.

In this work, carbonized corn-straw particles were combined with scleroglucan to construct a bio-based particle-polymer composite mobility-control system. The objectives were to: (i) prepare micron-scale carbonized biomass particles suitable for porous-media transport; (ii) evaluate the suspension stability and rheological response of the particle-scleroglucan system under different temperature and salinity conditions; (iii) assess its long-term aging stability under sealed anaerobic conditions; and (iv) determine its injectivity, plugging performance, and oil-displacement efficiency using sand-pack experiments. The study provides a basis for developing greener profile-control and oil-displacement technologies for harsh reservoir environments.

## 2. Materials and Methods

### 2.1. Experimental Materials

#### 2.1.1. Experimental Sample

The curdlan-producing strain used in this study is a Sclerotium rolfsii strain previously screened and preserved in our laboratory. It is stored at the National Key Laboratory for Enhanced Oil Recovery, China University of Petroleum, under ultra-low temperature conditions at −80 °C. Previous studies have shown that this strain exhibits a fast growth rate and stable fermentation yield, making it suitable for subsequent research on curdlan fermentation process optimization and oil displacement performance evaluation.

#### 2.1.2. Main Reagents for the Culture Medium

Potato Dextrose Agar (PDA) medium was prepared using 200 g of peeled potatoes, 20 g of glucose, and 15 g of agar, brought to a final volume of 1000 mL with water and adjusted to natural pH [24]. The seed medium contained (g/L) glucose 30, yeast extract 3, potassium dihydrogen phosphate 2, potassium chloride 1, ferrous sulfate 0.5, magnesium sulfate 0.5, and citric acid 1.0, while the fermentation medium contained (g/L) glucose 50, sodium nitrate 3, potassium dihydrogen phosphate 2, potassium chloride 0.5, ferrous sulfate 0.25, magnesium sulfate 0.5, and citric acid 1.0. All media were dispensed into culture containers and sterilized by autoclaving before use.

#### 2.1.3. Preparation of Crude Oil and Simulated Formation Water for Experiments

The simulated formation water had a salinity of 220 g·L^−1^, with detailed parameters listed in Table 1. The simulated oil was prepared by mixing dehydrated crude oil from the Qizhong area of Xinjiang with kerosene at a 2:1 ratio, exhibiting a viscosity of 2.75 mPa·s at 76.6 °C and a shear rate of 7.34 s^−1^.

### 2.2. Experimental Methods

#### 2.2.1. Instruments

The reagents used included absolute ethanol, sodium hexametaphosphate, NaCl, and CaCl2, which were purchased from Shanghai Macklin Biochemical Technology Co., Ltd. (Shanghai, China). Instruments employed were a fluorescence optical microscope (ZEISS, Oberkochen, Germany), mechanical grinder (Shanghai Bilang Instrument Co., Ltd., Shanghai, China), carbonization furnace (Luoyang Juwei Instrument Equipment Co., Ltd., Luoyang, China), planetary ball mill (Changsha Miqi Instrument Equipment Co., Ltd., Changsha, China), laser particle size analyzer (Malvern Panalytical, Malvern, UK), elemental analyzer (Elementar, Langenselbold, Germany), ultrasonic disperser (Kunshan Ultrasonic Instrument Co., Ltd., Kunshan, China), HAAKE rheometer (Thermo Fisher Scientific, Waltham, MA, USA), high-speed centrifuge (Beckman Coulter, Brea, CA, USA), electric blast drying oven (Shanghai Yiheng Scientific Instrument Co., Ltd., Shanghai, China), and displacement apparatus (Haian Fada Petroleum Instrument Technology Co., Ltd., Nantong, China).

#### 2.2.2. Pretreatment of Biomass Raw Material

Corn stalks were cleaned to remove surface impurities and air-dried under ambient conditions for two days. The dried stalks were ground for 5 min using a mechanical grinder and then sieved through a 200-mesh screen. The obtained powder was further ground for 5 min and transferred to a carbonization furnace. Under a nitrogen atmosphere, the temperature was increased to 500 °C at a heating rate of 20 °C/min and maintained for 2.5 h. The pyrolyzed material was then cooled to room temperature to obtain carbonized biomass particles [24].

For ball milling, 25 g of carbonized particles was placed in a milling jar with 250 g of zirconia balls, and 100 mL of water was added. The milling speed was set to 400–500 rpm, and the particles were milled for 0.5, 1, 2, or 4 h. After milling, the samples were dried at 50 °C to obtain ball-milled carbonized biomass particles.

#### 2.2.3. Characterization of Biomass Particles

Particle-size analysis was performed to evaluate whether the prepared biomass particles had a suitable size range for injection and migration in porous media. Approximately 1.0 g of the carbonized particle sample prepared as described above was dispersed in 50 mL of deionized water. A few drops of 0.5 wt% sodium hexametaphosphate were added as a dispersant to ensure thorough and stable dispersion. The particle size distribution of the suspension was then measured using a laser particle size analyzer. Before each measurement, the samples were ultrasonically dispersed for 3 min to break up particle agglomerates. The refractive index of the sample was set to 1.52, the dispersant (water) to 1.33, and measurements were repeated three times at room temperature.

Elemental analysis was conducted to assess the carbonization degree and the elemental composition of the carbonized biomass particles. Samples were dried to constant weight under vacuum at 60 °C to completely remove residual moisture. About 2.0 mg of dried, homogenized sample was placed in a tin capsule, compacted, and fed into a combustion tube via an automatic sampler. Under carbonization temperature (950 °C) and an oxygen-rich atmosphere, the sample underwent instantaneous combustion and oxidation. The resulting gas mixture was converted in a reduction tube, separated using an adsorption–desorption column, and finally detected by a thermal conductivity detector.

Suspension experiments were conducted to evaluate the dispersion stability and sedimentation resistance of the particle–polymer system. Carbonized particle samples were dispersed in 30 mL of deionized water or hard polysaccharide (curdlan) solutions with concentrations of 500 ppm, 1000 ppm, 1500 ppm, 2000 ppm, 2500 ppm, and 3000 ppm, following different solid-to-liquid ratios (carbonized particles: curdlan). The mixtures were placed in 25 mL graduated test tubes with stoppers and manually shaken once per second for 2 min to achieve uniform dispersion, then allowed to settle for 48 h. Measurements of the supernatant height were recorded every 12 h. After settling, the height of the upper clear liquid and the total suspension height were recorded to calculate the suspension rate. Samples with 0.1 wt% particle concentration were also tested in curdlan solutions of different concentrations to evaluate suspension capacity, with the suspension rate used as the assessment criterion. The calculation formula for the suspension rate is shown in Equation (1).

*Q* = (*h*_1_ − *h*_2_)/*h*_1_ (1)

*Q* is the suspension rate (%);

*h*_1_ is the total height of the suspension (cm);

*h*_2_ is the height of the supernatant after settling for 48 h (cm).

#### 2.2.4. Preparation of the Mobility Control System

In this study, scleroglucan was produced from a Sclerotium rolfsii strain using the fermentation method reported in our previous work, followed by ethanol precipitation, centrifugation, drying, and grinding to obtain the polysaccharide powder [25].

Scleroglucan is a neutral extracellular β-glucan produced by Sclerotium species. Its chemical structure is composed of a β-(1→3)-D-glucopyranose main chain with single β-(1→6)-D-glucopyranosyl side groups attached to approximately every third backbone residue. This branched structure promotes the formation of a rigid triple-helical conformation in aqueous solution, which contributes to its excellent thickening ability and salt tolerance [25].

A 100 mL aliquot of the original fermentation broth was used to determine the curdlan content via alcohol precipitation. The broth was mixed with three volumes of absolute ethanol, allowed to stand for precipitation, and then centrifuged. The precipitate was collected and dried at 60 °C to a constant weight to calculate the actual curdlan content in the fermentation broth. Based on this measurement, an appropriate volume of the broth was taken, sequentially centrifuged (10,000 rpm, 10 min) and filtered through a 0.45 μm microporous membrane to remove cells and insoluble impurities. Three volumes of absolute ethanol were slowly added to the supernatant with stirring to fully precipitate curdlan. The resulting brown flocculent precipitate was collected by centrifugation, dried to constant weight in a vacuum oven at 50 °C, and then ground to obtain curdlan powder. The powder was dissolved in deionized water and heated in a water bath at 50 °C with magnetic stirring for 4 h. After stirring, the solution was allowed to rest for 2 h to remove bubbles, yielding the curdlan solution, whose viscosity was subsequently measured.

A total of 0.1 g of carbonized ball-milled biomass particles was dispersed in 50 mL deionized water with stirring at 300 rpm for 1 min. The mixture was then treated in an ultrasonic disperser for 10 min at 40 °C and 70% power to break up soft aggregates. After ultrasonication, the solution was stirred again at 300 rpm for 1 min, and the curdlan solution was slowly added. The mixture was further stirred for 10 min to obtain the particle–gel composite system, after which the apparent viscosity was measured.

#### 2.2.5. Rheological Evaluation Under Different Temperature and Salinity Conditions

The salinity and temperature tolerance of the system were evaluated mainly by monitoring changes in apparent viscosity, storage modulus (*G*′), loss modulus (*G*″), and complex viscosity under different brine and temperature conditions. When designing high-temperature tests, a special rotor for high-temperature testing of the Hack rheometer is used to prevent moisture evaporation during the testing process. Curdlan solutions (1000 ppm) were prepared using deionized water, saturated NaCl solution, and saturated CaCl_2_ solution as solvents. Carbonized particles and the curdlan solutions were mixed at a 1:1 solid-to-liquid ratio. An aliquot of the equilibrated sample was placed in a HAAKE rheometer and subjected to a linear heating ramp from 30 °C to 130 °C at 2 °C/min. The temperature was held constant for 3 min every 10 °C increment, while the apparent viscosity at a shear rate of 7.34 s^−1^ was continuously recorded. Within the linear viscoelastic region, a shear stress of 100 mPa and oscillation frequency of 1 Hz were applied to observe changes in storage modulus (*G*′) and loss modulus (*G*″) [26]. The temperature-ramping rheological test was conducted within a short duration at each temperature point to minimize water evaporation, and the obtained data were used to evaluate the short-term rheological stability of the system.

The salinity tolerance of curdlan under various mineralization conditions was studied. Solutions containing 1%, 5%, 10%, 25%, and saturated concentrations of NaCl or CaCl_2_ were used instead of deionized water to prepare 1000 ppm curdlan solutions. Carbonized particles were mixed with the saline curdlan solutions at a 1:1 mass ratio to form composite suspensions. Steady-shear tests were conducted on a HAAKE rheometer at 25 °C, recording the apparent viscosity at a constant shear rate of 7.34 s^−1^. Oscillatory tests were conducted over a frequency range of 0.01–10 Hz at a shear stress of 100 mPa to observe changes in *G*′, *G*″, and complex viscosity. Additionally, a strain sweep from 0.01 to 1000 mPa at 1 Hz was performed to further evaluate viscoelastic behavior.

Systems with different salinities were placed in 50 mL vials filled with 25 mL of solution. After evacuation, nitrogen gas was introduced to remove oxygen from both the vial and dissolved in the solution; this vacuum–nitrogen cycle was repeated three times to ensure an oxygen-free environment. The vials were then sealed and aged at 100 °C for 28 days. Samples were taken every 7 days to monitor changes in viscosity [27].

#### 2.2.6. Evaluation of Particle Migration Performance

The average pore-throat diameter of the sand-packed columns was estimated using the Kozeny–Carman equation to evaluate the matching relationship between particle size and pore structure [28].

Quartz sand with 50-, 100-, and 150-mesh sizes was mixed in specific proportions and packed into three sand-packed columns with permeabilities of approximately 553, 1235, and 2064 mD, respectively (see Table 2). The columns were evacuated with a vacuum pump for 4 h and then saturated with formation water (simulated formation water, 220 g/L salinity) for 12 h. After saturation, the column mass was measured to calculate porosity and pore volume. The injection rate was set at 0.5 mL/min and the temperature at 80 °C. Following primary water flooding to stabilize pressure, the particle–water solution was injected, and changes in pressure and effluent volume were recorded.

#### 2.2.7. System Sealing Performance Evaluation

A total of four artificial sand-packed models were prepared, including two columns with gas permeabilities of approximately 2000 mD and two columns with gas permeabilities of approximately 3000 mD. The detailed parameters are listed in Table 3. Each model was placed in a core holder, evacuated, and saturated with formation water with a salinity of 220 g/L. Initial water flooding was performed at a constant flow rate of 0.5 mL/min at 80 °C until the inlet pressure stabilized, and the stabilized pressure difference was recorded as *Δp_w_*. Subsequently, 1 pore volume (PV) of the particle suspension system was injected at the same flow rate, and the pressure difference during particle injection was recorded as *Δp_p_*. Secondary water flooding was then conducted until the pressure stabilized again, and the stabilized pressure difference was recorded as *Δp_rw_*. The resistance factor (*F_R_*), residual resistance factor (*F_RR_*), and plugging efficiency (*η*) were calculated as follows:

(2) $$F_{R}=\frac{\Delta p_{p}}{\Delta p_{w}}$$

(3) $$F_{RR}=\frac{\Delta p_{rw}}{\Delta p_{w}}$$

(4) $$\eta =\left(1-\frac{\Delta p_{w}}{\Delta p_{rw}}\right)\times 100\%$$

*Δp_w_*—Pressure after a primary water drive, MPa;

*Δp_p_*—pressure after biopolymer flooding, MPa;

*Δp_rw_*—pressure after subsequent water flooding, MPa.

#### 2.2.8. Evaluation of Oil Displacement Performance

Based on the results of the plugging performance evaluation, two artificial sand-packed columns with gas permeabilities of approximately 3000 mD were prepared, with detailed parameters listed in Table 4. After evacuation, saturation with formation water and oil, and aging, injections were performed according to the previously described procedure. Following primary water flooding to stabilize the pressure, 1 PV of a 1000 ppm curdlan solution and a particle–polymer suspension (curdlan 1000 ppm, carbonized particles 2000 ppm) were injected into the respective columns. During injection, changes in pressure, oil recovery, and water cut were recorded.

The experimental oil was a simulated high-temperature, high-salinity Middle Eastern reservoir oil, prepared by blending dehydrated crude oil from the Qizhong area of Xinjiang with kerosene at a ratio of approximately 2:1. The resulting simulated oil had a viscosity of approximately 2.75 mPa·s, measured at 76.6 °C and a shear rate of 7.34 s^−1^.

## 3. Results

### 3.1. Particle Size and Elemental Analysis of Biomass Particles

**Figure 1:**
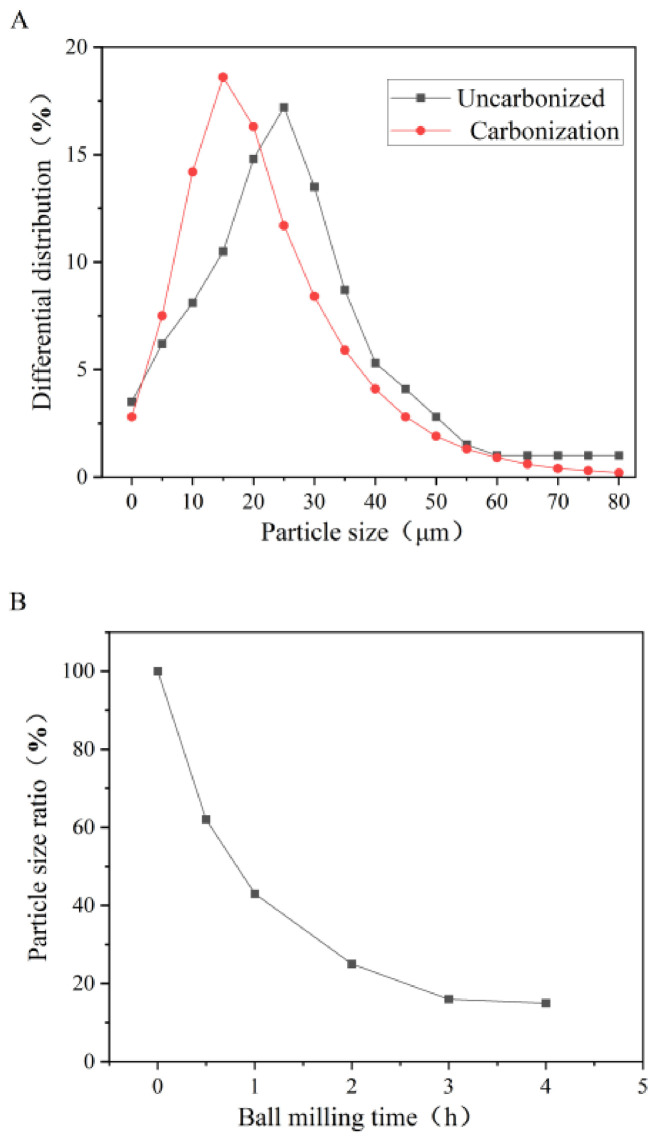
Particle size distribution after ball milling. (**A**): Particle size distribution of two types of particles after ball milling; (**B**): proportion of reduction in particle size of carbonized particles with ball milling time.

After carbonization, corn stalks subjected to further grinding exhibited a significant reduction in particle size and increased particle fineness [29]. The average particle size of mechanically ground, non-carbonized stalks was 25.6 μm, whereas that of the carbonized particles was 11.2 μm, with the size distributions shown in Figure 1A. These results indicate that carbonization increases the brittleness of the stalks, making them easier to break under mechanical shear, thereby significantly reducing particle size and making them more suitable for injection into reservoirs.

Ball milling further reduced the size of the carbonized particles. After 0.5 h of milling, the average particle size decreased from 11.2 to 6.9 μm, corresponding to a 38% reduction. After 2 h, the average particle size reached 2.8 μm, representing an overall reduction of approximately 75%. Prolonging the milling time beyond 2 h produced only limited additional size reduction, suggesting that the remaining finer particles required greater energy to overcome interparticle cohesion and further fragmentation. Considering both particle-size optimization and energy consumption, 2 h was selected as the optimal ball-milling time.

The elemental composition of the carbonized particles is summarized in Table 5. The carbon content was 42.08%, and the H/C atomic ratio was approximately 0.31. The low H/C ratio indicates an enhanced degree of aromatization after pyrolysis at 500 °C, which is consistent with the formation of a more thermally stable carbonaceous framework. The presence of inorganic elements such as K and P also suggests an appreciable ash fraction. These mineral components may influence particle density, surface charge, and interactions with brine ions; therefore, their effects on long-term reservoir compatibility should be considered in future work.

### 3.2. Screening of Suspension Stability of Biomass Particles

**Figure 2:**
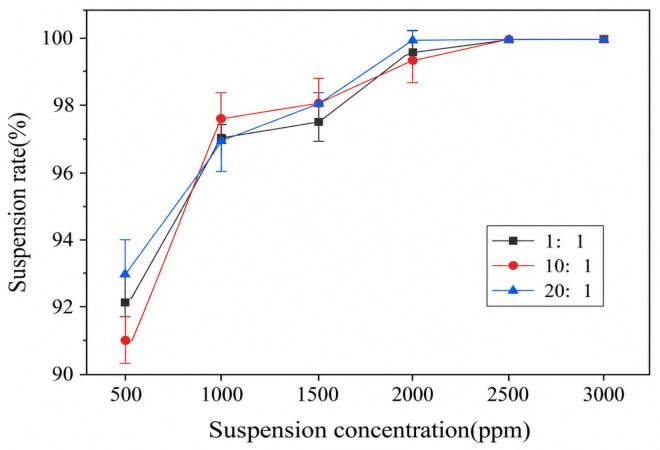
Variation Chart of Suspension Rate with Different Solid–Liquid Ratio.

Evaluating the effect of polymers on particle suspension is helpful for assessing the dispersion stability and sedimentation resistance of the mobility control system under reservoir conditions. Good suspension stability can reduce particle settling and blockage risks, ensuring smooth injection and deep migration of the system, thereby improving profile control, water plugging efficiency, and oil recovery [30]. The suspension results of different solid-to-liquid ratios (carbonized particles: curdlan) in suspensions of varying polymer concentrations are shown in Figure 2. The results indicate that the suspension ratio increases with both the concentration of the polymer solution and the particle concentration. When the polymer concentration increased from 500 ppm to 1000 ppm, the system exhibited the largest increase in suspension ratio, reaching 97% at 1000 ppm. For particle-based profile-control systems, a suspension rate above 90% is generally considered favorable because it indicates that most particles remain dispersed during the injection-relevant settling period, reducing the risk of premature sedimentation and near-wellbore blockage. Therefore, the suspension rate of 97% at 1000 ppm indicates favorable suspension stability while maintaining a relatively low polymer dosage. At polymer concentrations above 2000 ppm, the suspension ratio remained at 100% within 48 h, indicating a fully suspended state. As the polymer concentration increases, the viscosity and density of the fluid increase, slowing particle settling and improving suspension stability. Considering both suspension performance and economic efficiency, a polymer concentration of 1000 ppm was determined to be optimal for the suspension solution.

### 3.3. Evaluation of Rheological Properties and Tolerance of the Mobility Control System

**Figure 3:**
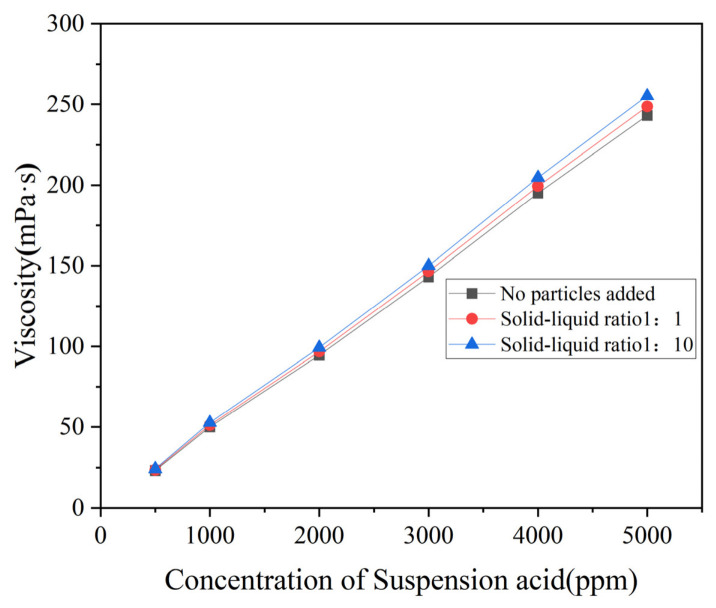
Relationship Diagram of System Concentration.

The relationship between the viscosity of the suspension system and the polymer concentration at different solid-to-liquid ratios is shown in Figure 3. The viscosity increases significantly with increasing curdlan concentration, while the addition of particles does not noticeably affect the system viscosity. The fitted correlation for the viscosity–concentration relationship without particles is expressed as y = 0.048x − 1.097 (where the independent variable x is the curdlan concentration and the dependent variable y is the viscosity). It is inferred that, within the current range of solid-to-liquid ratios, the particles mainly exist as the dispersed phase and contribute minimally to the continuous phase viscosity. Therefore, the system viscosity is still primarily governed by the entanglement and hydration of curdlan molecular chains. Overall, curdlan can maintain relatively stable flow behavior while ensuring particle suspension, providing a solid foundation for subsequent mobility control applications.

### 3.4. Thermal Stability Evaluation

What we are evaluating is the relationship between the temperature and viscosity of the suspended system, not the pyrolysis temperature. The stability of the system viscosity under high-temperature conditions is beneficial for maintaining good flow control, improving the swept volume, and enhancing the mobilization of residual oil, thereby increasing oil recovery efficiency and the ultimate recovery factor [30].

**Figure 4:**
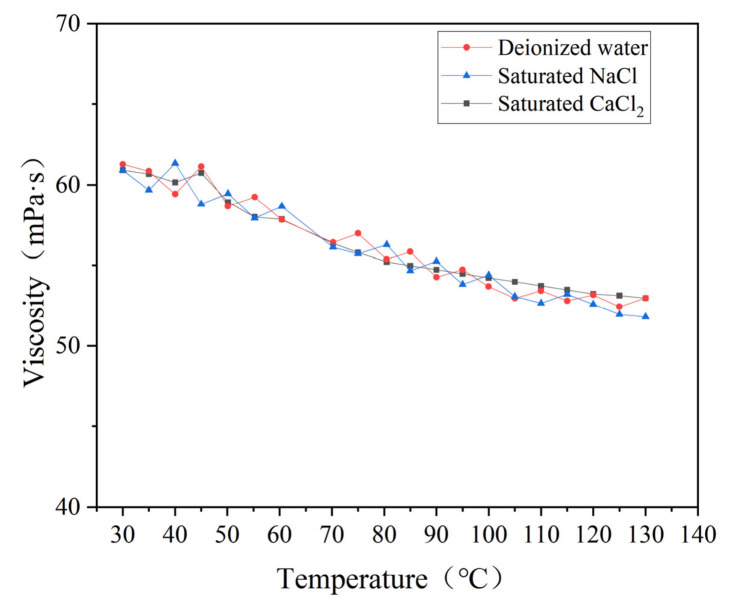
Graph showing the change in viscosity of the system at different temperatures.

The viscosity variation of the suspension systems at different temperatures is shown in Figure 4. Across the temperature range of 30 °C to 130 °C, the viscosity of the suspension systems gradually decreased with increasing temperature. At 130 °C, the viscosity loss of all three systems was less than 10% compared with their values at 30 °C. These results indicate that the suspension systems exhibit good thermal tolerance in formation waters with different ion types, enabling better adaptability to the high-temperature conditions of reservoirs.

**Figure 5:**
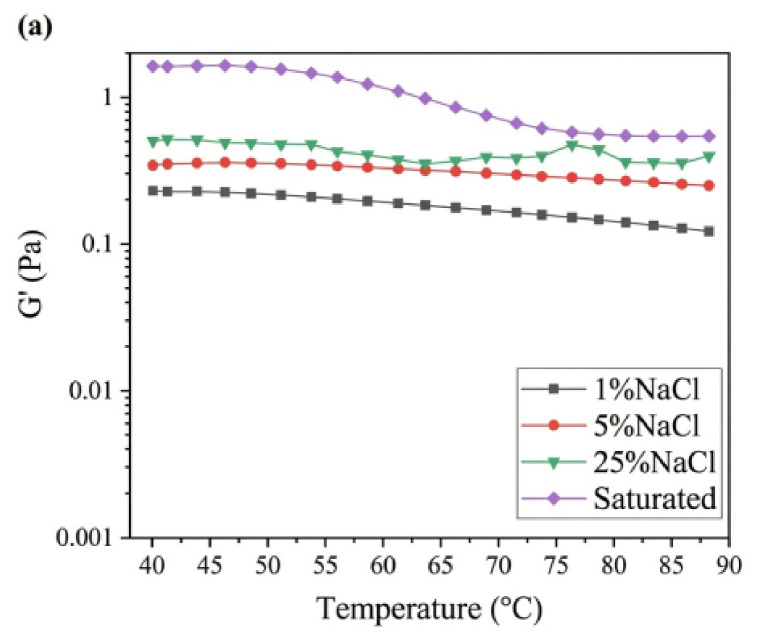

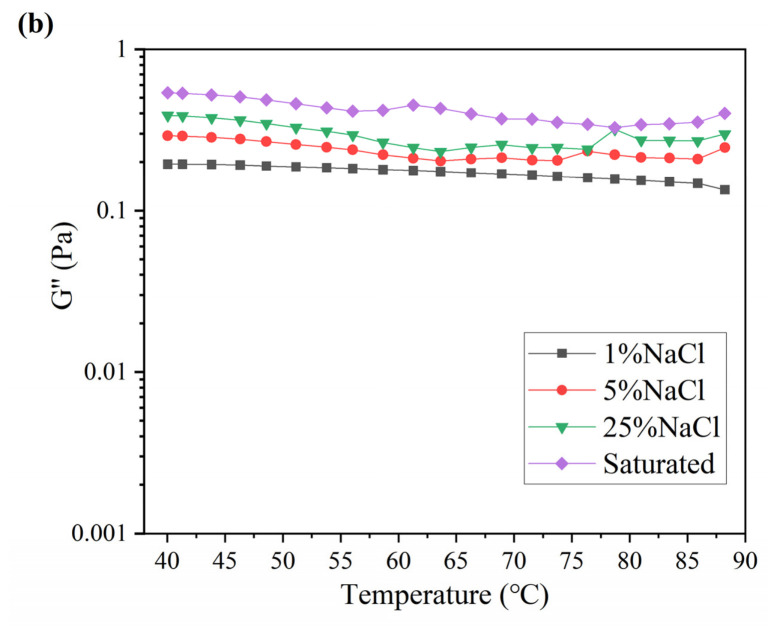
Graph showing the variations in G′ (**a**) and G″ (**b**) in the NaCl salt solution at different temperatures.

A higher *G*′ indicates a stronger elastic network within the system, which is beneficial for particle suspension and plugging performance. *G*″ reflects the viscous energy dissipation capacity, contributing to flow regulation in porous media. The stability of both *G*′ and *G*″ under high-salinity and high-temperature conditions suggests that the internal structure of the system is resistant to disruption. As shown in Figure 5, under different NaCl brine conditions, both the storage modulus (*G*′) and loss modulus (*G*″) remained within the same order of magnitude and changed only slightly with increasing temperature. With increasing NaCl concentration, *G*′ and *G*″ generally increased, indicating that salt concentration plays a more direct role in enhancing the viscoelastic response of the system. This result suggests that the system can maintain a stable internal network under high-salinity and high-temperature conditions [31].

**Figure 6:**
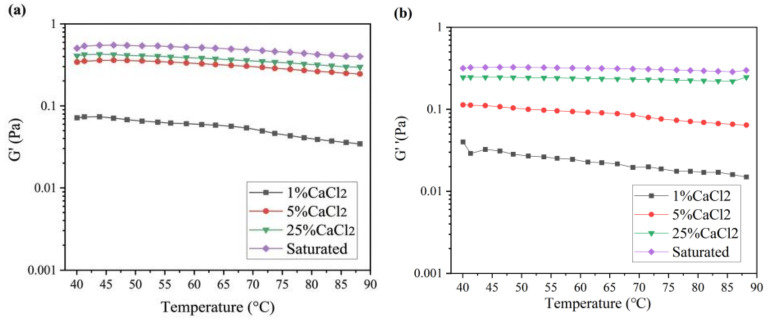
Variations in storage modulus *G*′ (**a**) and loss modulus *G*″ (**b**) of the system in CaCl_2_ solutions with different concentrations at different temperatures.

The variations in storage modulus G′ and loss modulus G″ of the system in CaCl_2_ solutions with different concentrations at different temperatures are shown in Figure 6. The relatively low *G*′ observed in the 1% CaCl_2_ system compared with the 1% NaCl system may be related to the different effects of monovalent and divalent ions on polymer-chain conformation and particle–polymer interactions. At low CaCl_2_ concentration, Ca^2+^ may partially screen intermolecular interactions and induce local chain contraction or localized particle–polymer association, which is insufficient to form a continuous elastic network. In contrast, 1% NaCl may favor a more homogeneous dispersion state and a weak but continuous network structure. With increasing CaCl_2_ concentration, the interaction between Ca^2+^ and the polymer–particle network becomes stronger, resulting in enhanced *G*′ and *G*″. Therefore, the lower *G*′ at 1% CaCl_2_ does not indicate poor salt tolerance, but reflects the concentration-dependent role of divalent ions in network formation. The rheological behavior of the system in CaCl_2_ solutions was generally similar to that observed in NaCl solutions. The values of *G*′ and *G*″ showed limited variation with increasing temperature, indicating good structural stability. Compared with Na^+^, Ca^2+^ produced a slightly stronger enhancement of the moduli, which may be related to the stronger interaction between divalent ions and the polymer–particle network. Nevertheless, the overall results indicate that the system exhibits good adaptability to both monovalent and divalent salt environments.

### 3.5. Evaluation of Salt Tolerance

The system was able to maintain relatively stable viscosity under different salinity conditions, indicating that its performance is minimally affected by variations in formation water mineralization. This characteristic helps maintain good flow control during displacement, expand the swept volume, and reduce viscosity loss and fluid channeling risks in high-salinity environments, thereby enhancing residual oil mobilization and improving oil recovery efficiency.

**Figure 7:**
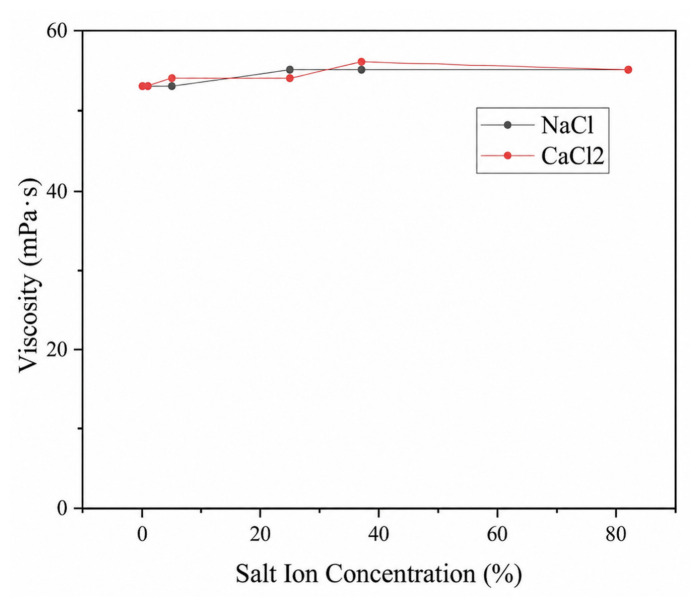
Curves showing the changes in system viscosity in solutions with different concentrations of NaCl and CaCl_2_ salts.

As shown in Figure 7, the apparent viscosity of the system remained relatively stable with increasing NaCl and CaCl_2_ concentrations, without significant viscosity loss. This indicates that the system has good salt tolerance. Compared with the difference between salt types, the effect of salt concentration on the rheological response was more evident. The stable viscosity in both NaCl and CaCl_2_ solutions demonstrates that the system can maintain its flow-control ability under different saline environments [32].

**Figure 8:**
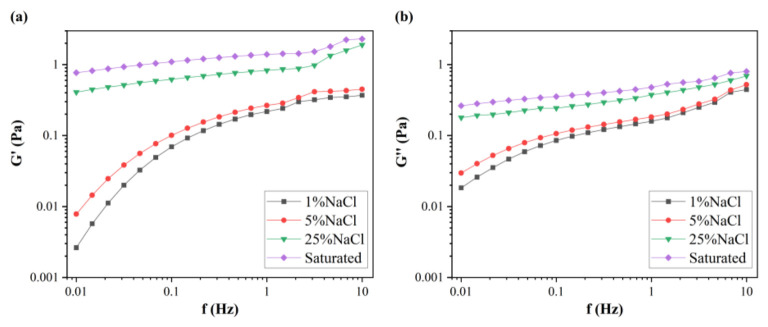
Curves of G′ (**a**) and G″ (**b**) changes under different frequencies in NaCl salt water.

**Figure 9:**
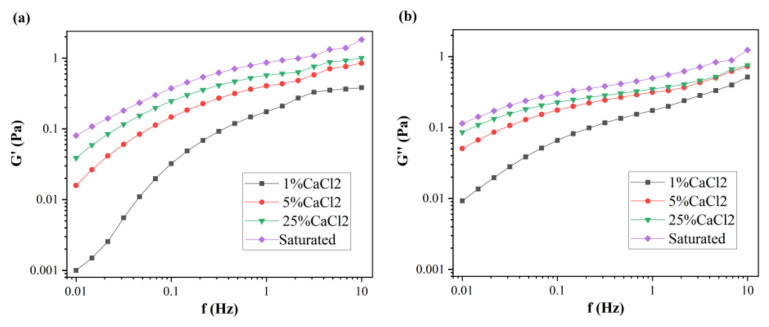
Changes in G′ (**a**) and G″ (**b**) curves under different frequencies in CaCl_2_ salt solution.

Investigating the effects of different ions on the rheological properties of the system helps to elucidate the viscoelastic response and structural stability under saline conditions, providing a theoretical basis for evaluating salt tolerance and reservoir adaptability. Figure 8 and Figure 9 show that *G*′ and *G*″ increased with frequency under both NaCl and CaCl_2_ conditions, and higher salt concentrations generally resulted in higher moduli. This indicates that increasing salinity promotes the formation of a more stable viscoelastic network. The enhancement in the CaCl_2_ system was slightly stronger than that in the NaCl system, suggesting that Ca^2+^ may contribute more effectively to network reinforcement. However, the overall trend was consistent in both salt systems, confirming the adaptability of the system to monovalent and divalent salts.

**Figure 10:**
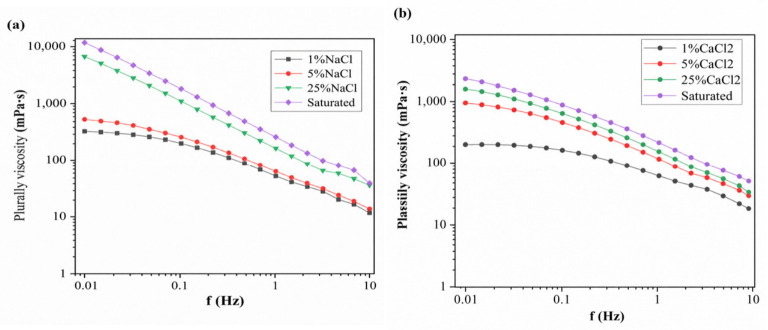
Complex viscosity variation curves at different frequencies in NaCl salt water (**a**) and CaCl_2_ salt water (**b**).

As shown in Figure 10, the complex viscosity decreased with increasing frequency in both NaCl and CaCl_2_ systems, indicating typical shear-thinning behavior. With increasing salt concentration, the overall complex viscosity tended to increase, suggesting that salinity enhances intermolecular interactions and particle–polymer network density. The similar variation trends in NaCl and CaCl_2_ solutions further indicate that the system maintains stable rheological behavior under different salt types.

**Figure 11:**
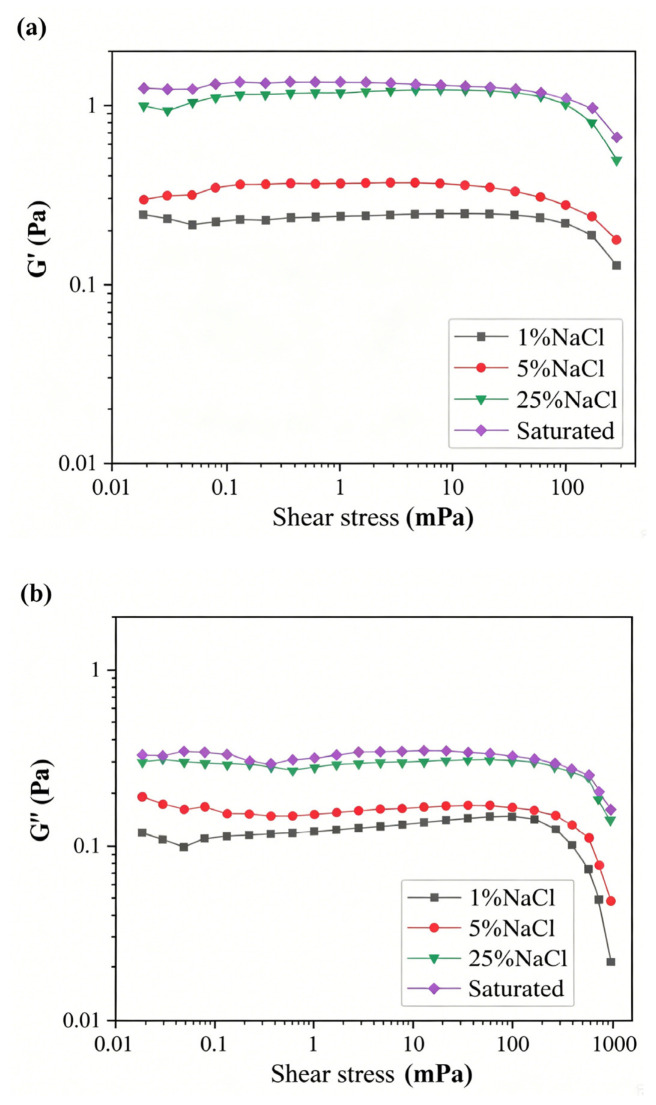
Curves of G′ (**a**) and G″ (**b**) changes under different shear stresses in NaCl salt water.

**Figure 12:**
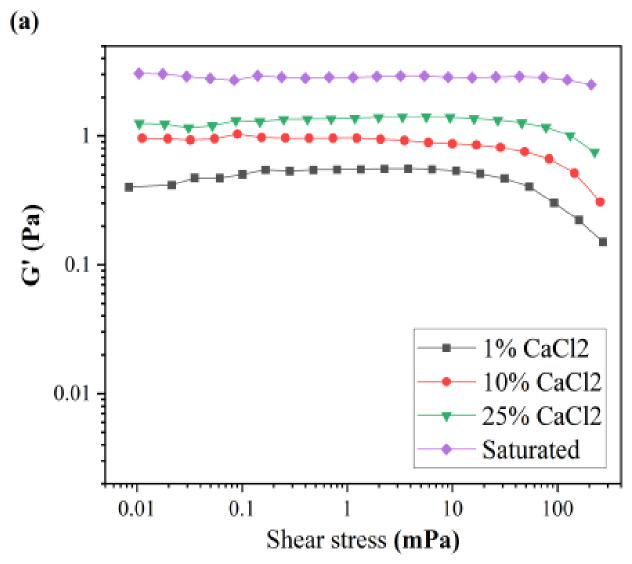

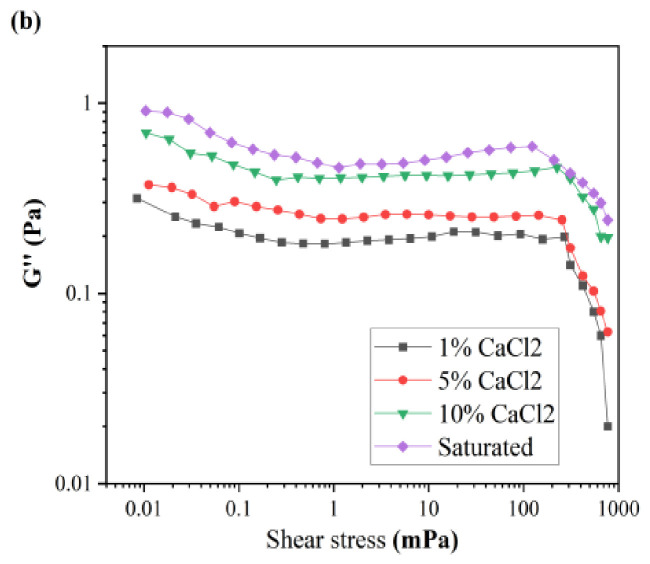
Variation curves of G′ (**a**) and G″ (**b**) in the CaCl_2_ system under different shear stresses.

Figure 11 and Figure 12 show that under both NaCl and CaCl_2_ conditions, *G*′ and *G*″ initially remained stable within the linear viscoelastic region and then decreased sharply after the critical stress was exceeded. Increasing salt concentration generally improved the moduli and broadened the linear viscoelastic region, indicating enhanced mechanical stability of the system. Ca^2+^ showed a slightly stronger reinforcement effect than Na^+^, but the system maintained good structural stability in both monovalent and divalent salt environments.

### 3.6. Long-Term Aging Stability Evaluation

Aging experiments of the suspension systems in different saline solutions were conducted under sealed, oxygen-free conditions at 100 °C to simulate high-temperature and high-salinity reservoir environments. This approach enables evaluation of the long-term thermal stability and salt tolerance of the systems, and investigation of the effects of different salt ion types on viscosity retention [33].

**Figure 13:**
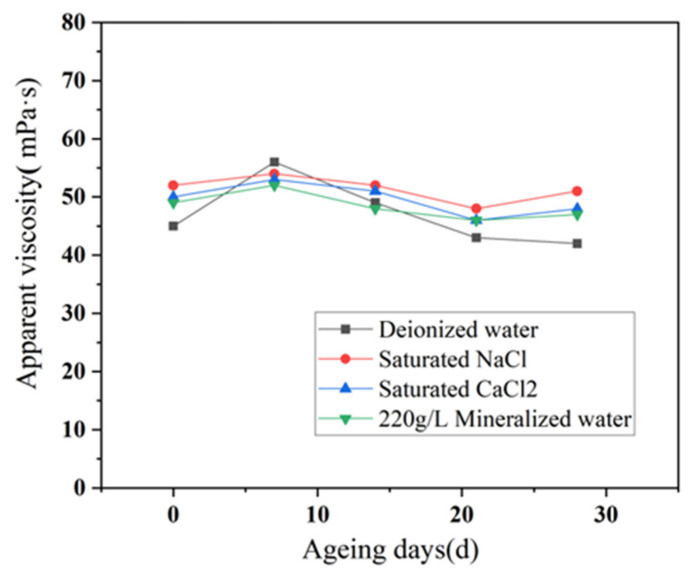
Curves showing the aging viscosity of the four suspension systems over 30 days as a function of aging time.

As shown in Figure 13, four suspension systems in different saline solutions retained over 90% of their initial viscosity after aging for 28 days under sealed, oxygen-free conditions at 100 °C. The salt-free system maintained essentially stable viscosity, whereas the saline systems exhibited a slight initial increase in viscosity followed by a gradual decline below the initial value. The initial increase is likely attributed to salt ions compressing the polymer chains, enhancing intermolecular interactions and reducing repulsive forces [34,35]. The subsequent decrease is presumably due to polymer chain entanglement and partial precipitation during aging, which reduces the soluble fraction and consequently lowers the overall viscosity [36].

### 3.7. Injectivity Evaluation

#### 3.7.1. Injectivity and Particle Migration in Sand-Packed Columns

Conducting injectivity evaluation studies helps to clarify the flow capacity and applicability range of the system in sand-packed tubes with different permeabilities, determine its feasibility for smooth reservoir injection, and provide a basis for subsequent evaluations of plugging and mobility control performance as well as optimization of field application parameters [37,38].

**Figure 14:**
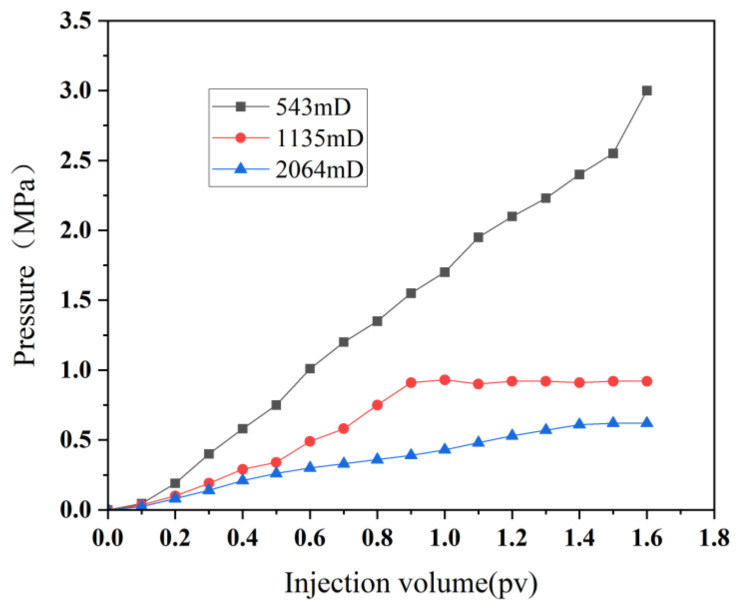
Pressure variation diagram of different permeability injection systems.

The pressure variation during the injection of the particle–scleroglucan suspension in sand-packed columns with different permeabilities is shown in Figure 14. The injection results are shown in Table 2. During injection into the 543 mD core, the pressure continued to increase and did not reach a stable state. Only a small amount of particles was observed in the produced fluid, and the produced fluid exhibited a light transmittance of 95%, indicating a low particle concentration because light transmittance is negatively correlated with particle concentration. In contrast, the produced fluids from the 1235 and 2064 mD cores showed light transmittance values of 24% and 0%, respectively, indicating that more particles migrated through the higher-permeability porous media. In contrast, the cores with permeabilities of 1235 mD and 2064 mD reached stable pressures after the injection of 1 PV and 1.3 PV, respectively. In particular, the light transmittance of the produced fluid from the 2064 mD core was 0%, indicating that more particles were able to migrate with the fluid to the end of the sand-packed tube. These results suggest that the system has good transport capacity and injectivity in high-permeability media, whereas its migration performance is relatively poor in low-permeability media [39].

(5) $$K=\frac{\phi \times r^{2}}{8\tau }$$

The stable pressure, light transmittance, and particle occurrence in the produced fluids are summarized in Table 6. According to the Kozeny–Carman equation, the estimated pore-throat diameters of the cores were 9.19 μm for B1, 13.28 μm for B2, and 15.20 μm for B3, respectively, with the tortuosity assumed to be 1.0. Taking the average particle size as 2.8 μm, the corresponding particle-to-pore-throat diameter ratios were 0.305, 0.211, and 0.184, respectively [28]. According to classical bridging theory, stable bridging is more likely to occur when the median particle size reaches approximately one-third of the average target pore-throat size; when the particle-to-pore-throat size ratio falls within the range of approximately 1/7–1/3, particles are more likely to enter the pore channels and undergo retention, local accumulation, and dynamic plugging [40]. Based on this, the 2.8 μm particles exhibited the highest size matching with the pore throats in the 543 mD model, with the size ratio approaching 1/3. Therefore, the particles were more likely to bridge and retain at pore-throat constrictions, causing local plugging, which was reflected by the continuously increasing injection pressure and the difficulty in reaching a stable pressure plateau. In the 1235 mD model, the particle-to-pore-throat size ratio decreased to 0.211, and the system was more likely to undergo a dynamic process of “entering pore channels—temporary local plugging—deformation or rearrangement—remigration”. As a result, it could generate a certain plugging resistance while maintaining relatively good subsequent transport capacity. In the 2064 mD model, the size ratio further decreased to 0.184, indicating that particles could more easily pass through the dominant pore throats and migrate deeper into the model. This resulted in a relatively low injection pressure differential and a faster transition to a stable state, suggesting stronger deep migration capacity. Therefore, based on this group of sand-packed model experiments, the 543 mD model was more prone to near-wellbore bridging and plugging, whereas the 2064 mD model favored deep migration and was more suitable for deep profile control and displacement. These results provide a basis for further evaluating the optimal permeability range applicable to the particle suspension system.

#### 3.7.2. Evaluation of Plugging Performance

The resistance factors, residual resistance factors, and plugging efficiencies of the different injection systems are summarized in Table 7. By comparing the plugging performance of the carbonized particle suspension with that of the pure polymer solution, the plugging effect of the carbonized particles can be clarified. The experimental results showed that the cores injected with polymer particles (B4 and B6) exhibited higher resistance factors (43.2 and 56.3) and residual resistance factors (23.6 and 41.2) than those injected only with polymer solution (B5 and B7, with resistance factors of 12.2 and 18.6 and residual resistance factors of 5.1 and 10.3, respectively). The plugging efficiencies reached 96.8% and 95.7%, which were superior to those of the control group (80.4% and 90.2%). These results indicate that the carbonized particles can form physical plugging through mechanical bridging, thereby further improving the plugging efficiency of the system [41]. Moreover, a more significant plugging enhancement effect was observed in the core with a permeability of 2846 mD.

#### 3.7.3. Oil Displacement Performance Evaluation

**Figure 15:**
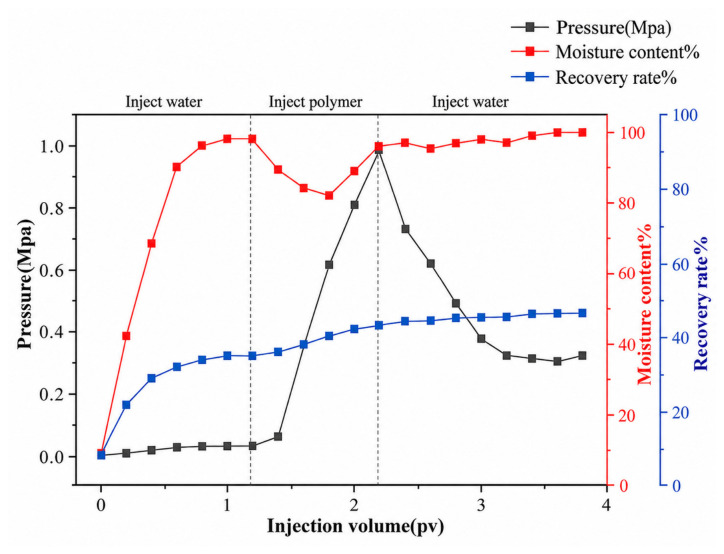

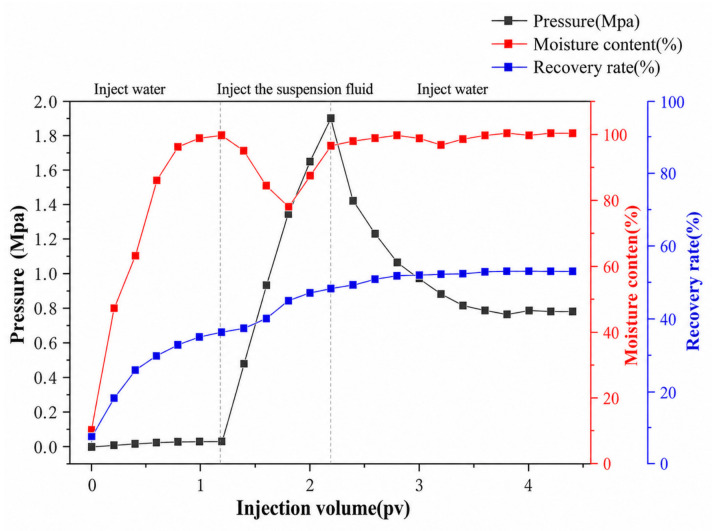
Curves of pressure, recovery factor, and water cut during polymer flooding (**top**) and carbonized particle suspension flooding (**below**).

Figure 15 compares the mobility control performance of the carbonized particle suspension and the pure polymer solution, showing clear differences in enhanced oil recovery behavior. The incremental oil recovery obtained by secondary flooding with the pure polymer was 12.5%, whereas the polymer–particle suspension achieved an incremental recovery of 16.7%. The total oil recovery of the carbonized particle suspension reached 53.3%, which was higher than the 46.6% achieved by polymer flooding alone. The incorporation of carbonized particles into the polymer system more effectively improved oil recovery. The particles enhanced the plugging and flow diversion capacities of the system while also reducing the required polymer dosage.

The resistance factor, residual resistance factor, and oil recovery results of the scleroglucan flooding and composite system are summarized in Table 8. The resistance factor increased from 30.8 for scleroglucan flooding to 56.2 for the composite system, while the residual resistance factor increased from 9.4 to 23.6. These increases indicate stronger flow resistance and more durable residual plugging after particle addition. The retained particles likely diverted subsequent water flow from dominant high-permeability channels into less-swept regions, thereby enlarging the swept volume and mobilizing additional residual oil [42]. Importantly, this improvement was achieved without increasing the scleroglucan concentration, suggesting a synergistic effect between carbonized particles and the biopolymer matrix.

## 4. Outlook

The biomass material used in this study was corn straw, and the suspending agent was the biopolymer scleroglucan. At present, several studies have reported the preparation of oil-displacement agents using biomass materials such as straw and biopolymers as raw materials. Wen Jialin [43] prepared straw ash particles from waste straw through carbonization, with an average particle size of approximately 300 nm. These particles increased oil recovery by 35.90% in a reservoir with a permeability of 500 mD.In this study, the biomass feedstock was obtained from agricultural waste corn straw and was converted into functional particles by carbonization at 500 °C followed by ball milling for 2 h. Under simulated high-temperature and high-salinity reservoir conditions in the Middle East, sand-pack flooding experiments showed that the biomass particle–scleroglucan composite system not only exhibited tolerance under extreme conditions, but also showed potential for profile control and oil displacement in high-permeability reservoirs, effectively improving oil recovery. In future work, the injectivity optimization of this system in low-permeability reservoirs will be further investigated.

Straw biomass can not only be used to prepare green profile-control and oil-displacement particles, but also has the potential to serve as a nutrient source for reservoir microorganisms and as a raw material for in situ gas generation [44]. Tian Huime [45] developed a thickened slow-release nutrient agent with both microbial activation and profile-control functions. At an optimal injection volume of 0.4 PV, the system significantly increased displacement pressure, improved oil recovery by up to 53.7% in single-core experiments, and enhanced oil displacement efficiency by 21.8% in parallel-core experiments. Wang Weidong [46] used green plant-derived materials to activate microbial oil recovery. In field trials, the maximum methane production reached 4.0 × 10^6^ m^3^, and oil recovery increased by more than 15%. As an agricultural solid waste, straw has renewable and low-carbon characteristics, which are consistent with the requirements of sustainable reservoir development. Through anaerobic fermentation, straw can generate gases such as methane and hydrogen, which help reduce crude oil viscosity and improve reservoir flow conditions, while also enabling partial recycling of carbon resources [47,48].

From the perspective of technical application prospects, the composite profile-control and oil-displacement system developed in this study provides a green and efficient development strategy for high-temperature and high-salinity reservoirs. It not only realizes the high-value utilization of agricultural waste but also reduces dependence on conventional chemical agents. In addition, the gas-generating potential of straw biomass can be integrated with profile-control and oil-displacement technologies, showing broad application prospects in the redevelopment of high-water-cut and near-depleted reservoirs [14].

## 5. Conclusions

1. Carbonization combined with ball milling effectively reduced the average particle size of corn-straw biomass from 25.6 to 2.8 μm. A ball-milling time of 2 h was selected as the optimal treatment condition. Elemental analysis showed a low H/C atomic ratio of 0.31, indicating enhanced aromatization and structural stability of the carbonized particles.
2. A scleroglucan concentration of 1000 ppm provided a suspension rate of approximately 97%, exceeding the favorable suspension criterion of 90% defined in this study. The particle–scleroglucan composite system maintained stable viscosity and viscoelasticity under reservoir-relevant high-temperature and high-salinity conditions. The viscosity loss remained below 10% during the short-term temperature-ramping test from 30 to 130 °C, and the viscosity retention remained above 90% after sealed anaerobic aging at 100 °C for 28 days, indicating excellent aging stability.
3. Sand-pack experiments demonstrated that the composite suspension had good injectivity in models with permeabilities of 1235 and 2064 mD, while the 543 mD model was more prone to near-inlet bridging and retention. Plugging tests showed that carbonized particles substantially increased the resistance factor, residual resistance factor, and plugging efficiency compared with scleroglucan alone.
4. In oil-displacement experiments, the particle-scleroglucan composite system increased the final recovery factor to 53.3%, which was 6.7 percentage points higher than that obtained by scleroglucan flooding alone. The improved performance was attributed to particle bridging, residual plugging, and flow diversion, which enlarged the swept volume and enhanced residual-oil mobilization.

## Figures and Tables

**Table 1:** Simulated brine composition.

Composition (g·L^−1^)			Total Salinity (g·L^−1^)
NaCl	CaCl_2_	MgCl_2_·6H_2_O	
192.5	16.5	11	220

**Table 2:** Parameters of Sandstone Cores for Injection Experiment.

Core ID	Diameter/cm	Length/cm	Pore Volume/cm^3^	Porosity/%	Gas Permeability/mD
B1	5	20	71.87	18.3	543
B2	5	20	86.79	22.1	1235
B3	5	20	110.74	28.2	2064

**Table 3:** Parameters of Sandstone Core for Plugging Experiment.

Core ID	Diameter/cm	Length/cm	Pore Volume/cm^3^	Porosity/%	Gas Permeability/mD
B4	5	25	132.95	27.1	2135
B5	5	25	128.35	26.2	2062
B6	5	25	141.30	28.8	3162
B7	5	25	129.03	26.3	3254

**Table 4:** Parameters of Sandstone Cores for Oil Displacement Experiments.

Core ID	Gas Permeability/mD	Water-Measured Permeability	Core Parameters		Pore Volume/cm^3^	Saturated Oil Volume (mL)
			Length/cm	Diameter/cm		
B8	3643	3056	20	2.5	70.2	48.6
B9	3254	2842	20	2.5	71.4	47.3

**Table 5:** Selected elemental composition of carbonized biomass particles. (The remaining fraction mainly includes oxygen-containing groups and inorganic ash/mineral components not individually quantified in this table).

Element	Content (%)
C	42.08
H	1.08
N	1.49
P	2.36
K	15.82
Others by difference	37.17

**Table 6:** Stable pressure, light transmittance and produced fluid of different permeability injection systems.

Core No.	Gas-Measured Permeability (mD)	Stable Pressure (MPa)	Light Transmittance of Produced Fluid (%)	Particles in Produced Fluid
B1	543	Not stabilized	95	No particles
B2	1235	0.92	24	Particles observed
B3	2064	0.62	0	Particles observed

**Table 7:** Resistance factors, residual resistance factors, and plugging efficiencies of injection systems in cores with different permeabilities.

Core No.	Gas-Measured Permeability (mD)	Water-Measured Permeability (mD)	Resistance Factor	Residual Resistance Factor	Plugging Rate (%)
B4	2135	1986	56.3	31.2	96.8
B5	2062	1860	18.6	10.3	90.2
B6	3162	2846	43.2	23.6	95.7
B7	3254	2856	12.2	5.1	80.4

**Table 8:** Effects of Different Permeabilities on Enhanced Oil Recovery, Resistance Factor, and Residual Resistance Factor.

Core No.	Water-Measured Permeability (mD)	Primary Waterflooding Recovery (%)	Secondary Waterflooding		Resistance Factor (RF)	Residual Resistance Factor (RRF)
			Incremental Recovery (%)	Total Recovery (%)		
B8	3056	34.2	12.5	46.6	30.8	9.4
B9	2842	36.6	16.7	53.3	56.2	23.6

## Data Availability

The original contributions presented in this study are included in the article. Further inquiries can be directed to the corresponding author.
